# Synergistic effects of biochar on soil organic carbon, greenhouse gas mitigation, and maize productivity in saline-alkali environments

**DOI:** 10.3389/fpls.2026.1774960

**Published:** 2026-03-25

**Authors:** Qian Qi, Haibin Shi, Jianwen Yan, Wenhao Ren, Liquan Fan, Yongde Su

**Affiliations:** 1 College of Water Conservancy and Civil Engineering, Inner Mongolia Agricultural University, Hohhot, China; 2 State Key Laboratory of Water Engineering Ecology and Environment in Arid Area, Inner Mongolia Agricultural University, Hohhot, China

**Keywords:** biochar, crop yield, greenhouse gas, saline-alkali soil, soil organic carbon

## Abstract

**Background and aims:**

Biochar is a widely recognized amendment for augmenting crop productivity and mitigating greenhouse gas (GHG) emissions, particularly in degraded soil. However, optimal biochar rate and their mechanisms remain uncertain across different salinity severities.

**Methods:**

To address this knowledge gap, a two crop seasons experiment was conducted from 2022 to 2023 on mildly (S1) and moderately (S2) saline soils, employing four biochar application rates: 0, 10, 20, and 40 t·ha^-1^. This study systematically evaluated the effects of biochar on crop yield, GHG emissions, and soil organic carbon.

**Results:**

The results revealed significant disparities in GHG emissions and carbon sequestration capacity between the two soil salinity levels. Under identical treatments, total soil CO_2_ emissions in S2 were 14.70%–26.99% lower than those in S1, whereas total soil N_2_O, CH_4_ emissions were elevated by 37.94%–61.04% and 34.41%–94.74%, respectively. For every 0.5 g·kg^-1^ increase in soil salinity, the soil organic carbon storage (SOCS) declined by 6.09%. Biochar application substantially increased crop yield (↑14.51%-30.65%) and SOCS (↑218.51%-404.52%) while concurrently mitigating global warming potential (GWP, ↓10.08%-24.12%), greenhouse gas intensity (GHGI, ↓20.75%-41.84%), and net greenhouse gas emissions (NGHGE, ↓205.83%-437.14%) (P< 0.01). Pearson’s correlation analysis demonstrated significant negative correlations between SOC/SOCS and GHG emission indicators as well as a strong relationship between crop yield and GHGI.

**Conclusion:**

Regression optimization modeling identified the optimal biochar application rates as 26.67 t·ha^-1^ for S1 and 30.82 t·ha^-1^ for S2, which achieved the most favorable balance between agricultural productivity and environmental benefits. This study demonstrates that appropriate biochar application can significantly enhance the carbon sink function of saline-alkali farmlands, reduce climate risks, and provide a promising strategy for sustainable agriculture in marginal lands.

## 1 Introduction

As the impact of climate change escalates and global food demand continues to rise, modern agriculture faces the critical imperative of enhancing production efficiency, while concurrently reducing its environmental footprint. Agricultural activities are a substantial source of greenhouse gas (GHG) emissions. In Asia, they contribute approximately 44% of the regional total, whereas globally, agri-food systems account for approximately 30% of anthropogenic GHG emissions (Vergé et al., 2007). The principal contributors to the agricultural GHG budget are nitrous oxide (N_2_O) and methane (CH_4_), which originate primarily from the application of nitrogen fertilizers and prevailing soil management practices (Stavi and Lal, 2012; Tubiello et al., 2013). Concurrently, saline-alkali lands, representing a significant reserve of potential arable resources, cover nearly 1 billion hectares worldwide, with China alone possessing over 100 million hectares (Xia-Yu et al., 2022; Yang et al., 2024a). This land has considerable potential for future agricultural development. However, saline-alkali soils are typically characterized by high pH, low organic matter content, and degraded physicochemical properties (Zhang et al., 2025). These conditions restrict crop growth and diminish yield (Xu et al., 2023) but also exacerbates environmental conditions, such as nitrogen loss and elevated GHG emissions (Li et al., 2024; Wang et al., 2023). Particularly under conventional irrigation and fertilization in these regions, frequent fluctuations in soil redox conditions create biogeochemical hotspots that intensify microbial processes, leading to increased emissions of gases such as N_2_O and carbon dioxide (CO_2_) (Wang et al., 2024c; Zhang et al., 2023b). Therefore, there is an urgent need to explore field management strategies that can simultaneously mitigate emissions and enhance yields to achieve green and efficient agricultural development on saline-alkali lands (Gan et al., 2011; Ogle et al., 2014).

Numerous studies have demonstrated that biochar, a stable and carbon-rich soil amendment, can alleviate salt stress by enhancing the cation exchange capacity and improving the soil aggregate structure (Sun et al., 2021; Wang et al., 2024b). Biochar application has also been shown to promote the accumulation of soil organic matter (Cong et al., 2023; Plaza et al., 2016) and improve nitrogen retention capacity, thereby increasing crop yield and nitrogen-use efficiency (Zheng et al., 2013). Furthermore, biochar can effectively suppress N_2_O emissions and enhance CH_4_ uptake (Chen et al., 2024; Shaukat et al., 2019). Consequently, biochar plays a significant role in reducing GHG emissions and strengthening soil carbon sequestration (Li et al., 2022; Wu et al., 2023b). In recent years, biochar has been widely applied in saline–alkaline soil improvement owing to its remarkable effects on soil physicochemical properties, nutrient retention, and microbial activity. However, excessive application of biochar may further increase soil pH and electrical conductivity, potentially limiting nutrient availability and crop growth (Moradi et al., 2019). Therefore, the application of biochar in saline–alkaline soils requires careful consideration of both the severity of soil salinity and the appropriate application rate. Nevertheless, there are notable gaps in this study. First, most investigations have focused on single salinity-alkalinity types or have been conducted under controlled laboratory conditions, with a lack of systematic field validation across different salinity and alkalinity gradients. Second, the key synergistic mechanisms through which biochar achieves the trifecta of “emission reduction-yield increase-carbon sequestration” in saline-alkaline soils remain unclear. Finally, optimal biochar application rates are often determined empirically and lack quantitative optimization methods and comparative studies across varying salinity and alkalinity conditions. Therefore, within the context of regionally heterogeneous saline-alkali lands, there is a pressing need to develop optimal biochar application strategies tailored to different salinity-alkalinity levels to achieve synergistic ecological and productivity benefits.

The Hetao Irrigation District is a typical Yellow River diversion irrigation area in China (Xiong et al., 2023; Xu and Song, 2022) characterized the region as having an arid and semi-arid climate and a widespread distribution of mildly to moderately saline-alkali soils (He and Liu, 2024; Zhu et al., 2022). It serves as an important commercial grain and forage production base in Northwest China (Yin et al., 2024; Yin et al., 2022). Long-term irrigation practices have induced secondary salinization, subjecting farmland in the region to the multiple stresses of a “water-salt-nitrogen” imbalance, which urgently requires green input measures to improve soil quality and crop productivity (Chang et al., 2019; Xiong et al., 2023). Maize, one of the principal crops in the region (Di et al., 2024), exhibits strong representativeness and high nitrogen responsiveness, making it a suitable indicator crop for assessing farmland nutrient management and carbon emissions (Huang et al., 2022; Yu and Shang, 2024). Concurrently, agricultural activities in the Hetao Irrigation District offer significant potential for regulating greenhouse gas emissions and soil carbon sequestration capacity, rendering it a representative area for studying the coupled effects of “emission reduction–yield increase–carbon sequestration” in farmland systems (Liu et al., 2023; Yang et al., 2024b). Biochar, a material possessing both stable and soil conditioning functions, has recently gained a foundation for its promotion in this region. Its application has practical significance in designing regional agricultural carbon neutrality pathways (Burrell et al., 2016; Qian et al., 2023).

To systematically evaluate the effects of biochar on emission reduction and yield enhancement in saline-alkali soils, this study conducted a two crop seasons experiment using typical mildly (S1) and moderately (S2) saline-alkali soils with four biochar application rates: 0, 10, 20, and 40 t·ha^-1^. The aims of this study were to 1) simultaneously monitor crop yield and greenhouse gas emissions; 2) investigate soil carbon indicators to elucidate the regulatory effects of biochar on carbon and nitrogen cycling and ecological processes in saline-alkali farmlands; and 3) apply a multi-objective response surface optimization approach to quantitatively determine the optimal biochar application rates under different salinity levels. This optimization sought to maximize yield, minimize global warming potential (GWP), and enhance soil organic carbon storage (SOCS), thereby developing an application strategy that balances productivity with environmental benefits. This study not only addresses the research gap regarding the synergistic response of “emission reduction-yield increase-carbon sequestration” from biochar application in saline-alkali soils but also proposes and validates a quantitative pathway for differentiated optimal application rates for the first time. These findings provide technical references for precise nutrient management in saline-alkali soils, efficient utilization of marginal lands, and enhancement of agricultural carbon sinks in arid regions. This study contributes to promoting green and low-carbon transformation of agriculture and achieving regional carbon neutrality goals, thereby demonstrating significant theoretical value and application potential.

## 2 Materials and methods

### 2.1 Experimental site description

**Figure 1:**
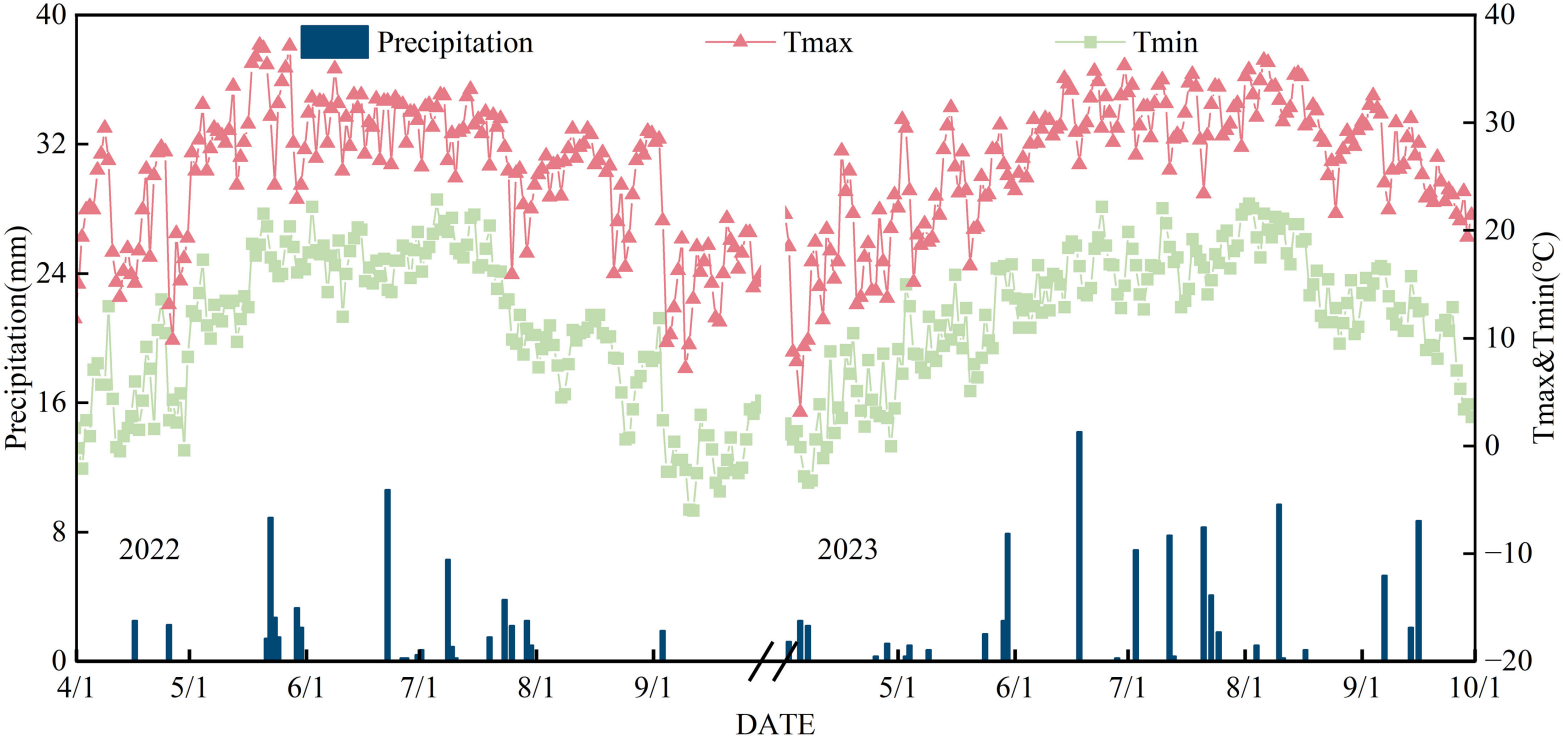
Rainfall, irrigation and temperature during maize growth period in 2022 and 2023.

The field experiment was conducted from April 2022 to October 2023 at the Science and Technology Backyard in Wuyuan County, Bayannur City, Hetao Irrigation District, Inner Mongolia, China (108°00′27″E, 41°04′39″N). The region is characterized by a temperate continental climate, with a mean annual temperature of 6.1 °C and a frost-free period ranging from 117 to 136 days. The average annual precipitation is 170 mm, occurring predominantly from June to August and constituting approximately 70% of the total annual rainfall in the region. The soil at the experimental site was classified as sandy loam. Prior to the experiment, the initial physicochemical properties of the topsoil (0–20 cm) in mildly and moderately saline farmlands were determined. For the mildly and moderately saline soils, respectively, the salt content was 1.34 g·kg^-1^ and 2.85 g·kg^-1^, pH was 7.75 and 8.15, organic matter content was 10.04 g·kg^-1^ and 9.56 g·kg^-1^, available nitrogen was 56.48 mg·kg^-1^ and 47.61 mg·kg^-1^, available phosphorus was 13.87 mg·kg^-1^ and 11.77 mg·kg^-1^, and available potassium was 132.33 mg·kg^-1^and 105.43 mg·kg^-1^. A meteorological station was installed on-site to facilitate the automatic recording of weather data throughout the experimental period (**Figure 1**).

### 2.2 Experimental design

The experiment was conducted at two soil salinity levels: mild (S1) and moderate (S2). The selected test crop was maize (Zea mays), cultivar “Xianyu 1321”. Sowing was performed on May 3, 2022, and April 28, 2023, with harvests occurring on September 23 and September 22, respectively. A conventional planting pattern of two rows per plastic film mulch was utilized, featuring a row spacing of 0.4 m and a plant spacing of 0.24 m, resulting in a planting density of 67,500 plants per hectare.

The experimental design incorporated four distinct biochar application rates for each of the two saline soil types: 0 t·ha^-1^ (T0), 10 t·ha^-1^ (T10), 20 t·ha^-1^ (T20), and 40 t·ha^-1^ (T40). This resulted in eight treatments, each replicated thrice in a randomized block design. Individual experimental plots were 64 m^2^ (8 m × 8 m). During the growing season, border irrigation was applied at the jointing, tasseling, and filling stages using water sourced from the Yellow River, with an irrigation quota of 750 m^3^·ha^-1^ per for each application. According to local agronomic practices, diammonium phosphate (DAP; containing 18% N and 46% P_2_O_5_) was applied as a basal fertilizer at a rate of 300 kg·ha^-1^, equivalent to 54 kg N·ha^-1^ and 138 kg P_2_O_5_·ha^-1^, during the film-mulching before sowing. Topdressing nitrogen was applied at 225 kg N·ha^-1^ using urea (46% N), split equally (1:1) at the jointing and tasseling stages. All other agronomic management practices were consistent with the conventional local farming protocols.

The biochar used in the experiment was procured from the Hengrui Huanke Company in Ordos City, Inner Mongolia. It was produced via pyrolysis of corn straw under anaerobic conditions at 360 °C. The resulting biochar had a pH of 8.7, carbon content (ω(C)) of 47.6%, nitrogen content (ω(N)) of 0.7%, and a C/N ratio of 68. Biochar was applied once as a uniform surface broadcast on the experimental plots before crop sowing in 2022 and was subsequently incorporated into the 0–20 cm soil layer with a rotary tiller. No additional biochar was applied in 2023, and field experiments were conducted *in situ* to assess residual effects.

### 2.3 Experimental methods

#### 2.3.1 Soil sampling and analysis

Soil samples were collected from three depths (0–20 cm, 20–40 cm, and 40–60 cm) within each treatment plot before maize sowing and after the final harvest. Following collection, the samples were air-dried in a cool, ventilated environment, crushed, and passed through a 2 mm sieve. Soil organic carbon (SOC) was determined using the potassium dichromate-concentrated sulfuric acid external heating method.

Soil organic carbon storage (SOCS) was calculated using the following formula (Equation 1) (Han et al., 2023):

(1) $$SOCS=\sum \left(SOC_{a}-SOC_{b}\right)\times BD\times H$$

where *SOCS* is the soil organic carbon storage (kg·ha^-1^), *SOC_a_* and *SOC_b_* are the initial and final SOC contents (g·kg^-1^) of the soil layer, respectively, *BD* is the soil bulk density (g·cm^-3^), and *H* is the thickness of the soil layer (cm).

#### 2.3.2 Greenhouse gas sampling and measurement

The greenhouse gas fluxes were measured using a static opaque chamber gas chromatograph. The sampling apparatus comprised a stainless-steel chamber (50 cm × 50 cm × 50 cm) and a corresponding base (50 cm × 50 cm × 50 cm). After maize sowing, the base was installed at the center of each plot to ensure an airtight seal between the soil and base groove. The chamber was insulated with a reflective material to minimize the influence of external environmental conditions on the internal microclimate during gas collection. Gas sampling was conducted between 09:00 and 12:00 h at 15-day intervals. During each sampling event, gas samples were withdrawn from the chamber headspace at 10-minute intervals over a 30-minute period (0, 10, 20, and 30 min). The temperature of the internal chamber was recorded at the beginning and end of the sampling period. The gas samples were analyzed using a Picarro G2308 analyzer.

The fluxes of N_2_O, CH_4_, CO_2_ were calculated as follows (Equation 2) (Jiang et al., 2021):

(2) $$f=\rho \times h\times \frac{dc}{dt}\times \frac{273}{273+T}$$

where f is the gas emission flux (mg·m^-^2·h^-^1), ρ is the gas density under standard conditions (kg·m^-^3), h is the height of the chamber (m); $\frac{dc}{dt}$ is the slope of the regression curve of gas concentration change over time (mg·h^-^1), and T is the average temperature inside the chamber during sampling (°C).

The cumulative emissions of N_2_O, CH_4_, CO_2_ were calculated using the following equations (Equation 3) (Wang et al., 2021):

(3) $$F=\sum_{i=1}^{n}\frac{f_{i}+f_{i+1}}{2}\times \left(t_{i+1}-t_{i}\right)\times 24$$

where *F* is the cumulative gas emission (kg·ha^-1^), *f* is the gas emission flux (mg·m^-2^·h^-1^); $t_{i+1}-t_{i}$ for the i-th sampling event, and *Di* is the number of days between two consecutive sampling events (d).

The global warming potential (GWP) was calculated as follows (Equation 4) (Lu et al., 2024):

(4) $$GWP=F_{CO_{2}}\times 1+F_{CH_{4}}\times 34+F_{N_{2}O}\times 298$$

where *GWP* is the global warming potential (kg·ha^-1^), and $F_{CO_{2}},F_{CH_{4}}$ $F_{N_{2}O}$ are the cumulative emissions of N_2_O, CH_4_, CO_2_ respectively (kg·ha^-1^).

Net greenhouse gas emissions (NGHGE) were calculated using the following formula (Equation 5) (Han et al., 2023):

(5) $$NGHGE=F_{CH_{4}}\times 34+F_{N_{2}O}\times 298-SOCS\times \frac{44}{12}$$

where *NGHGE* is the net GHG emission (kg·ha^-1^).

The greenhouse gas intensity (GHGI) was calculated as follows (Equation 6) (Ma et al., 2021):

(6) $$GHGI=\frac{GWP}{Yield}$$

where *GHGI* is the greenhouse gas intensity (kg·kg^-1^) and *Yield* is the crop yield (kg·ha^-1^).

#### 2.3.3 Maize yield

At physiological maturity, ten maize plants exhibiting similar growth characteristics were randomly selected from each of the plots. Following manual threshing and air-drying of the grains, the 100-grain weight was determined and the grain yield was subsequently calculated.

### 2.4 Data analysis

Data processing and statistical analyses were performed using Microsoft Excel 2024 and SPSS Statistics 26.0 (IBM, USA). The experimental plots were considered as independent experimental units, and each treatment was replicated three times (n = 3). One-way analysis of variance (ANOVA) followed by the Least Significant Difference (LSD) test was employed to compare the mean differences among treatments. Statistical significance was set at p< 0.05. Pearson correlation analysis was performed to examine the relationships among grain yield, greenhouse gas emissions, and soil organic carbon. All figures were generated using the Origin 2024 software.

## 3 Results

### 3.1 Greenhouse gas emission characteristics

#### 3.1.1 CH_4_ emission flux

**Figure 2:**
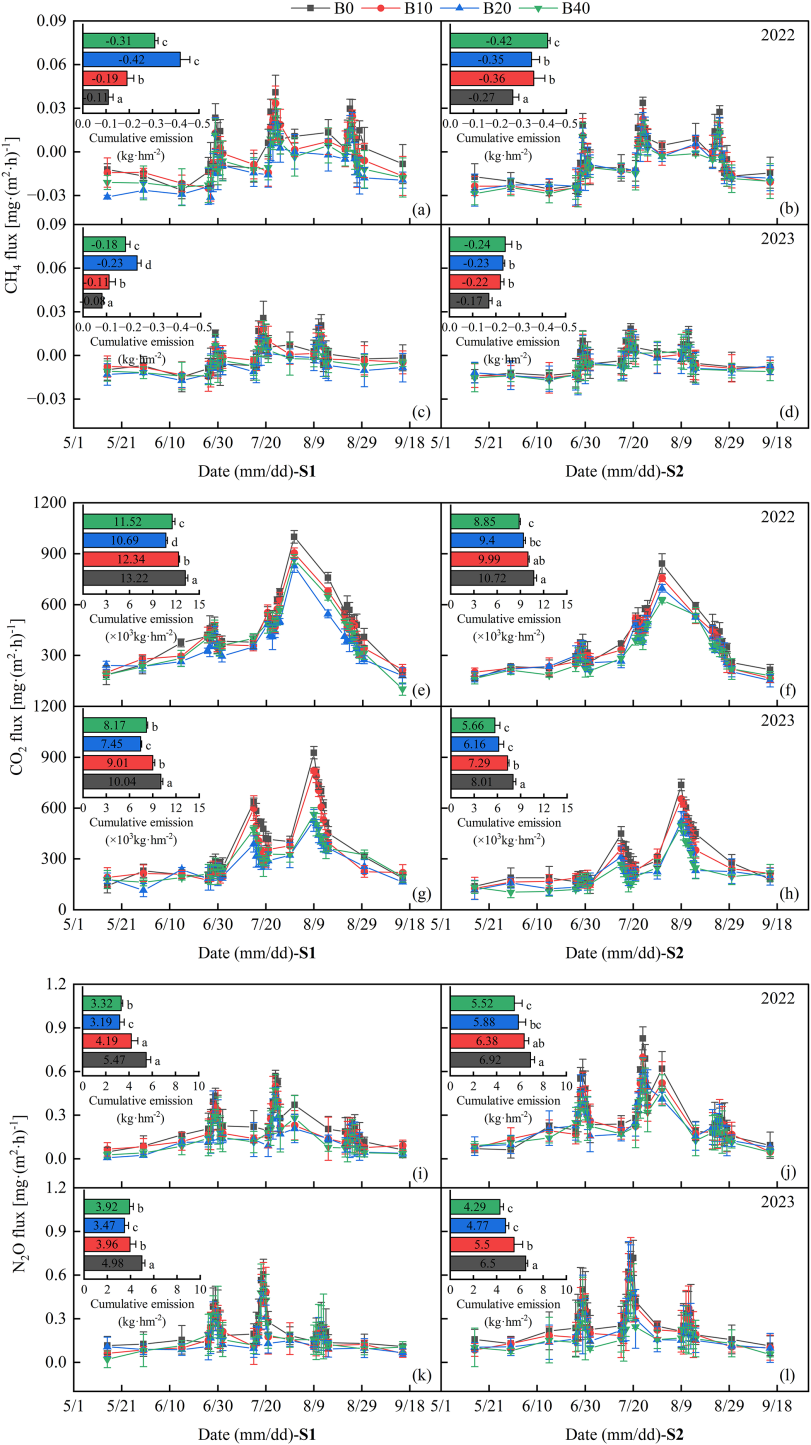
Effects of biochar application rate on seasonal CH₄, CO₂, and N₂O fluxes under different saline conditions during 2022–2023. S1 and S2 denote slightly saline and moderately saline soils, respectively. Panels **(a–d)**, **(e–h)**, and **(i–l)** represent CH₄, CO₂, and N₂O fluxes, respectively, for S1 and S2 in 2022 and 2023; the inset in each panel shows cumulative emissions.

The temporal dynamics of CH_4_ emission fluxes across the different treatments during the 2022 and 2023 maize growing seasons in saline farmlands are shown in **Figures 2a, b, g, h**. Positive and negative values indicate CH_4_ emission and absorption, respectively. Pronounced fluctuations in the flux were observed after each irrigation event.

In 2022, under mildly saline (S1) conditions, the capacity of biochar to enhance soil aeration and promote methanotrophic activity contributed significantly to CH_4_ absorption in, the B20 and B40 treatments, which exhibited average fluxes of -0.003 mg·m^-2^·h^-1^ and -0.002 mg·m^-2^·h^-1^, respectively. Compared to the control (CK), the average peak CH_4_ emission fluxes in these treatments decreased by 50.74% and 47.64%, respectively. Under S2 conditions, the average CH_4_ fluxes for all treatments ranged from -0.003 to -0.0009 mg·m^-2^·h^-1^, with the B40 treatment exhibiting the most substantial absorption. The average peak CH_4_ flux in S2 was 0.008 mg·m^-2^·h^-1^, which was value 0.56 times that recorded in S1. In 2023, the B20 and B40 treatments demonstrated the lowest CH_4_ emission flux. Relative to CK, the average peak fluxes were reduced by 44.31% and 48.47%, respectively. The enhanced mitigation effect observed in 2023 was primarily attributed to the lagged influence of biochar on the microbial activity.

Throughout 2022, all the treatments functioned as net sinks for atmospheric CH_4_. The lowest cumulative CH_4_ emissions under S1 and S2 were recorded in the B20 and B40 treatments. In comparison to CK, these represented reductions of 186.47% and 40.44%, respectively. A similar pattern was observed in 2023. Under S1, the cumulative emission of CK was -0.11 kg·ha^-1^, whereas the B20 treatment enhanced absorption to -0.42 kg·ha^-1^. In moderately saline-alkali soils (S2), characterized by stronger ionic stress, a biochar application rate of 40 t·ha^-1^ (B40) was required to achieve a comparable level of CH_4_ absorption. These findings indicated that appropriate biochar application can effectively promote soil CH_4_ uptake. Over the two crop seasons period, at equivalent biochar application rates, S1 treatments significantly diminished CH_4_ absorption by 24.33%–53.70% compared to S2 treatments, suggesting that moderately saline-alkali soils have a greater potential for CH_4_ absorption.

#### 3.1.2 CO_2_ emission flux

During the maize growing season, the CO_2_ emission fluxes for all treatments exhibited multipeak dynamics (**Figures 2c, d, i, j**). The primary emission periods were concentrated during vigorous growth stages of tessellation and filling. In 2022 and 2023, the average CO_2_ emission fluxes ranged from 323.15–478.81 mg·m^-2^·h^-1^ and 226.40–427.59 mg·m^-2^·h^-1^, respectively. Biochar application mitigated CO_2_ emissions, with decreases of 6.97%–19.46% and 7.70%–18.39% under S1 and S2 conditions in 2022, respectively. In 2023, the corresponding reductions were 13.11%–31.79% and 9.34%–29.72%.

Under the S1 conditions, the rank order of the average peak CO_2_ emission fluxes across both years was CK > B10 > B40 > B20. This pattern may be attributed to excessive biochar application, which enhances soil aeration and creates favorable conditions for microbial activity, thereby accelerating the mineralization of soil organic carbon. In 2022 and 2023, the B20 treatment yielded average peak fluxes of 663.68 mg·m^-2^·h^-1^ and 457.76 mg·m^-2^·h^-1^, representing reductions of 20.55% and 41.39% compared to CK, respectively. Under S2 conditions, the B40 treatment resulted in the lowest average peak CO_2_ emissions, followed by B20, with average reductions of 28.48% and 22.72% over the two crop seasons, respectively, relative to CK. At equivalent biochar application rates, the peak CO_2_ emission fluxes in S1 consistently surpassed those in S2, indicating that elevated soil salinity could attenuate CO_2_ emissions.

Under S1 conditions, cumulative CO_2_ emissions initially decreased and then increased with increasing biochar application rates. Compared to CK, the average reductions for B20 and B40 were 22.47% and 15.73%, respectively. This suggests that excessive biochar application may increase the total CO_2_ emissions in mildly saline spring maize fields. In 2022, under S2 conditions, the cumulative CO_2_ emission in CK was 10,720.28 kg·ha^-1^, which was significantly reduced to 8,845.71 kg·ha^-1^ in the B40 treatment, a decrease of 17.49%. A significant reduction of 29.33% was observed by 2023. Overall, the results suggest that biochar can effectively mitigate CO_2_ emissions, likely by enhancing soil carbon sequestration capacity.

#### 3.1.3 N_2_O emission flux

Significant peaks in N_2_O emission fluxes were registered across all treatments 4–5 d following fertilization or irrigation events, as illustrated in **Figures 2e, f, k , l**). Notably, elevated flux periods were observed during late June and mid-July. Under S1 conditions in 2022, the application of biochar mitigated the average N_2_O emission flux by 18.28%–36.40% relative to the control (CK) treatment. The peak average N_2_O emission flux recorded for the B20 treatment measured 0.28 mg·m^-2^·h^-1^, representing a 31.29% reduction compared to CK. Under the S2 conditions, the overall emission trend followed the sequence CK > B10 > B20 > B40. The average peak N_2_O emission fluxes for the B20 and B40 treatments were 0.28 and 0.24 mg·m^-2^·h^-1^, respectively.

In 2023, the average N_2_O emission fluxes under S1 and S2 conditions ranged from 0.19–0.25 mg·m^-2^·h^-1^ and 0.22–0.33 mg·m^-2^·h^-1^, respectively. fluxes were marginally higher in 2023 than those in 2022, which was principally attributed to the natural aging of biochar *in situ*. This aging process reduced soil permeability, subsequently promoting enhanced adsorption and inhibitory effects. Furthermore, peak emissions were registered slightly earlier in 2023 than in 2022, influenced by the adjusted timing of irrigation and fertilization events.

The capacity of biochar to enhance water and nitrogen retention effectively decreases nitrogen loss during nitrification-denitrification processes. Consequently, biochar application reduced cumulative N_2_O emissions by 7.82% to 41.76% compared with the CK treatment across the two crop seasons. Significant mitigation effects were observed in the B20 and B40 treatments under the S1 and S2 conditions, yielding average reductions of 36.07% and 27.08%, respectively. At identical biochar application rates, cumulative N_2_O emissions under S2 increased by an average of 37.94%–61.04% relative to S1. This increase primarily stems from the inhibition of N_2_O reductase activity during denitrification induced by elevated salt ion concentrations. A comprehensive assessment indicated that a suitable biochar application effectively suppressed greenhouse gas emissions. The 20 t·ha^-1^ and 40 t·ha^-1^ application rates exhibited stable performance under S1 and S2 conditions, respectively. The emission reduction efficacy under S2 was particularly pronounced, thereby substantiating the feasibility of implementing biochar application strategies in moderately saline- alkaline soils.

### 3.2 Effects of different biochar application rates on GWP, GHGI, NGHGE, and yield

The results presented in **Table 1** demonstrate that biochar application exerted a significant influence on crop yield within saline farmlands (P< 0.01). Over the two crop seasons (2022 and 2023), the yield exhibited a substantial increase commensurate with elevated biochar application rates, specifically from B0 to B20. Conversely, a further increase in the application rate of B40 failed to elicit additional yield enhancements, resulting in a marginal decline. This outcome was attributed to the potential of excess biochar to diminish soil nutrient availability, thereby constraining crop productivity. Generally, across both salinity levels, an optimum biochar application rate (B20) augmented yield by 15.67%–54.11%, confirming a significant yield-enhancing effect.

**Table 1:** Effects of biochar application rate on yield, 100-grain weight, Global Warming Potential (GWP), Greenhouse Gas Emission Intensity (GHGI), and Net Greenhouse Gas Emissions (NGHGE) under different saline conditions (S1: slightly saline; S2: moderately saline) during 2022–2023.

Year	Treatment		Yield (×10^3^kg·ha^-1^)	100-grain weight(g)	GWP (×10^3^kg·ha^-1^)	GHGI (kg·kg^-1^)	NGHGE (×10^3^kg·ha^-1^)
2022	S1	B0	13.10 ± 1.56c	32.36 ± 2.33ab	14.85 ± 0.21a	1.13a	22.50 ± 3.85a
		B10	14.95 ± 1.64b	30.55 ± 1.67b	13.58 ± 0.06ab	0.91b	-60.51 ± 6.07b
		B20	17.93 ± 1.82a	35.46 ± 1.91a	11.63 ± 0.11c	0.65c	-83.27 ± 4.83c
		B40	17.11 ± 1.84a	33.89 ± 2.39ab	12.50 ± 0.37bc	0.73c	-64.33 ± 5.15b
	S2	B0	12.50 ± 0.68c	31.74 ± 2.73b	12.77 ± 0.27a	1.22a	37.98 ± 3.19a
		B10	14.13 ± 1.08b	34.25 ± 2.84ab	11.88 ± 0.14ab	0.98b	-40.87 ± 3.66b
		B20	15.32 ± 1.08a	32.38 ± 1.97ab	11.14 ± 0.38bc	0.73c	-59.45 ± 4.38c
		B40	16.45 ± 1.07a	35.37 ± 2.7a	10.48 ± 0.25c	0.64c	-62.27 ± 4.24c
2023	S1	B0	14.22 ± 0.69b	30.98 ± 2.13c	11.52 ± 0.20a	0.81a	20.64 ± 3.16a
		B10	15.18 ± 1.20ab	35.98 ± 2.43ab	10.18 ± 0.11b	0.67b	-52.38 ± 1.70b
		B20	16.44 ± 1.42a	37.2 ± 2.68a	8.48 ± 0.07bc	0.52c	-62.78 ± 3.57c
		B40	16.07 ± 0.93a	33.38 ± 3.52bc	9.34 ± 0.14c	0.58c	-55.57 ± 5.01b
	S2	B0	12.18 ± 0.78c	32.5 ± 2.01b	9.94 ± 0.40a	0.98a	28.03 ± 2.31a
		B10	14.34 ± 0.63b	35.94 ± 3.15ab	8.92 ± 0.41b	0.72b	-29.16 ± 3.67b
		B20	15.68 ± 0.80a	36.29 ± 2.48a	7.58 ± 0.61c	0.48c	-53.30 ± 3.74c
		B40	15.44 ± 1.02a	36.82 ± 2.33a	6.93 ± 0.63bc	0.45c	-59.23 ± 3.09d
ANOVA							
S(Salt)	—		**	ns	**	**	*
B(Biochar)	—		**	ns	**	**	**
S×B	—		ns	ns	**	**	**

During the maize growth cycle, CO_2_ emissions are the primary determinant of Global Warming Potential (GWP). Relative to the control (CK), biochar implementation consistently mitigated GWP, Greenhouse Gas Intensity (GHGI), and Net Greenhouse Gas Emissions (NGHGE) within the salinized agricultural system. The most pronounced mitigation effects were documented for the B20 treatment under S1 and B40 treatments under S2. These treatments reduced GWP by 24.07% and 24.12% and GHGI by 39.60% and 50.85%, respectively. At an equivalent nitrogen application rate, the GWP in S2 was 7.41%–20.98% lower than that in S1. These findings suggest that biochar application conferred more reliable emission reduction advantages in moderately saline-alkali soils, thereby effectively reducing total greenhouse gas emissions and improving the environmental efficiency of agricultural production.

ANOVA indicated that the interaction between soil salinity and biochar application rate exhibited a highly significant impact on GWP, GHGI, and NGHGE (P< 0.01); however, this interaction did not significantly influence yield (ns). This demonstrates that the effectiveness of biochar in mitigating carbon emissions is substantially modulated by soil salinity levels, while the observed yield-enhancing effect remained comparatively robust and less sensitive to salinity. In summary, the adoption of an optimal biochar application rate (B20) within saline-alkali regions successfully augmented crop yields and concurrently achieved significant reductions in carbon emission intensity and net carbon emissions from agricultural practices.

### 3.3 Effects of biochar application on SOC and SOCS

**Figure 3:**
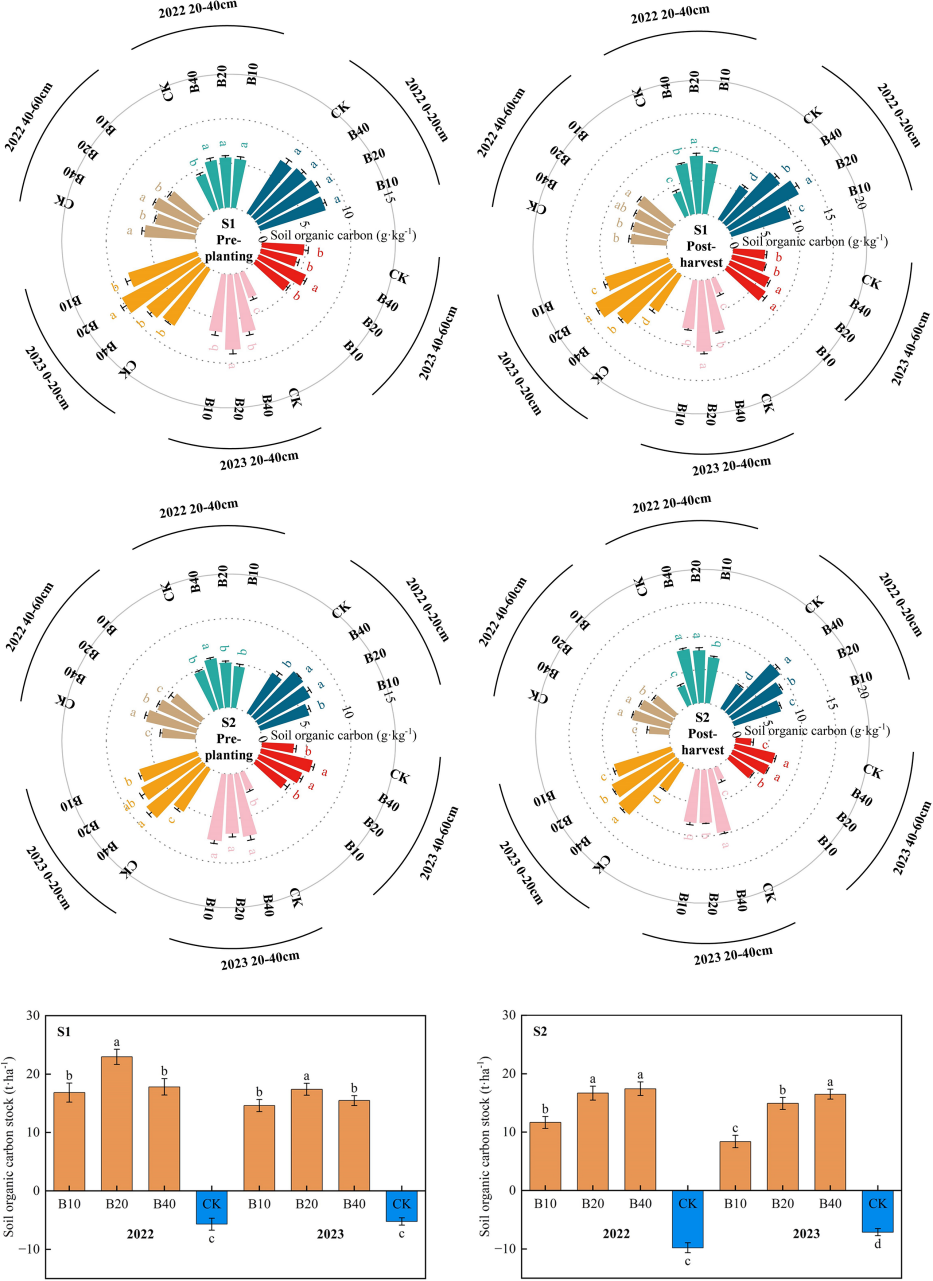
Soil organic carbon (SOC) content and soil organic carbon sequestration (SOCS) in response to different biochar application rates under different saline conditions (S1, slightly saline; S2, moderately saline) during 2022-2023.

The application of biochar substantially augmented both the soil organic carbon (SOC) content and soil organic carbon storage (SOCS) within the 0–60 cm soil profile across all salinity levels (**Figure 3**). Specifically, this enhancement was most pronounced within the 0–20 cm soil layer throughout the two crop seasons. At the time of the final harvest, the SOC content in mildly saline-alkali soil (S1) followed the hierarchical sequence: B20 > B40 > B10 > CK. Conversely, in the moderately saline-alkaline soil (S2), the observed sequence was B40 > B20 > B10 > CK. This disparity suggests that under S2 conditions, elevated salt stress potentially diminishes the ameliorative efficacy of biochar when applied at lower rates, necessitating a higher application rate (B40) to effectively elevate SOC levels.

Soil organic carbon storage (SOCS) was significantly augmented by biochar treatments. Over the two crop seasons, biochar application notably improved SOCS. Under S1 conditions, the B20 treatment yielded the maximum SOCS, registering an average increase of 25.64 t·ha^-1^ compared to CK and a 20.70% increase relative to the B40 treatment. Under S2 conditions, the B40 treatment achieved the highest SOCS, surpassing the B20 treatment by 7.51%; meanwhile, the B20 treatment increased SOCS by an average of 24.27 t·ha^-1^ relative to CK. Furthermore, at identical biochar application rates, SOCS under S2 conditions was diminished by 2.26%–36.63% compared to S1 conditions. These observations demonstrate that biochar application constitutes a highly effective strategy for enhancing soil carbon storage and boosting SOC, revealing particularly significant long-term improvement potential within moderately saline-alkali soils. In summary, appropriate biochar utilization substantially improves the soil organic carbon status in saline-alkali regions, thereby contributing to desirable agroecological outcomes and environmental sustainability.

### 3.4 Correlation analysis of yield, greenhouse gas emission indicators, and soil carbon pool changes

**Figure 4:**
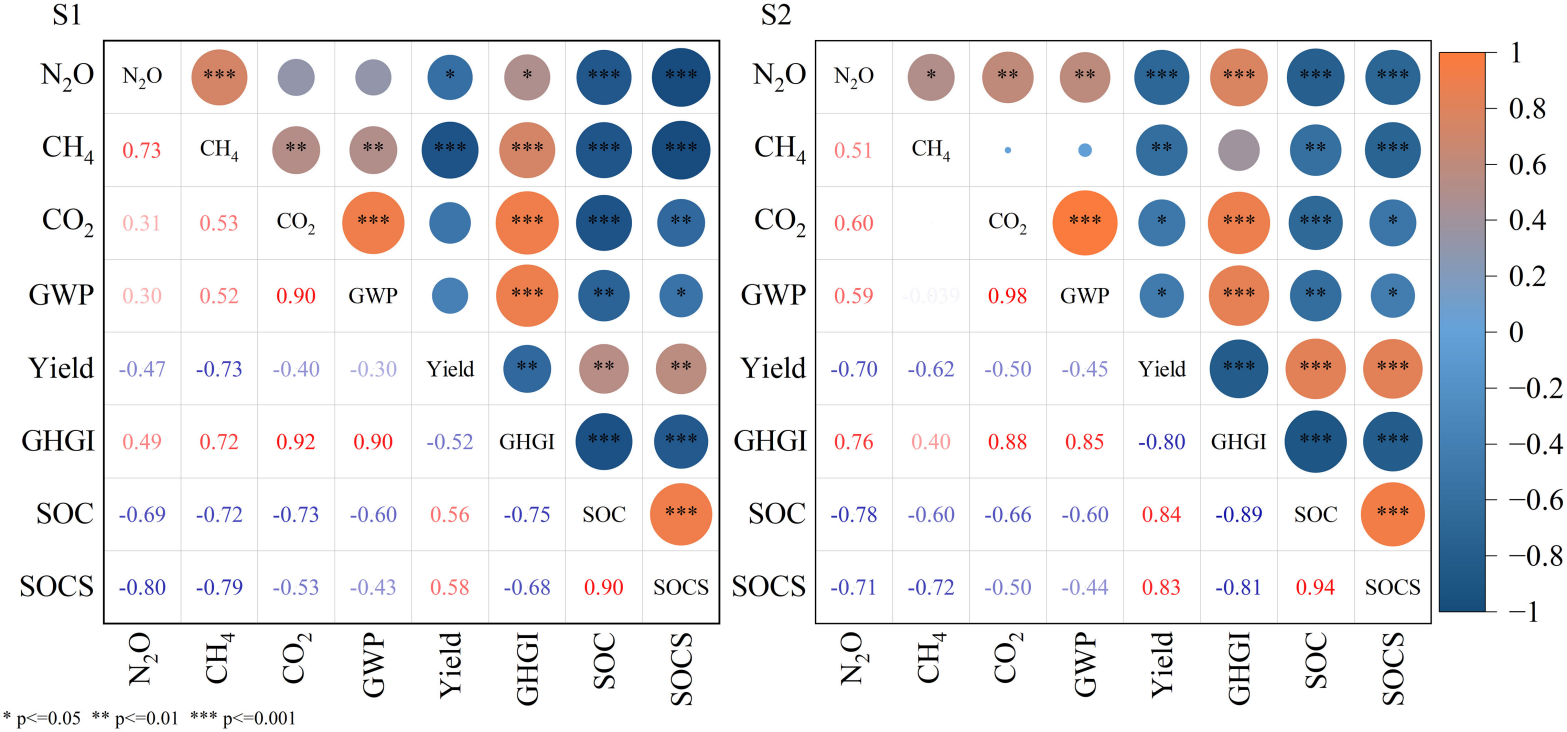
Pearson correlation analysis of yield, greenhouse gas emissions, and soil organic carbon indicators under different saline conditions (S1, slightly saline; S2, moderately saline) during 2022-2023.

Correlation analysis demonstrated that crop yield (yield) maintained a significant inverse relationship with various greenhouse gas emission indicators (P< 0.05), a pattern that was notably more pronounced in moderately saline-alkali soil (S2) (**Figure 4**). The strongest inverse association was observed between Yield and Greenhouse Gas Intensity (GHGI) (r = -0.80, P< 0.001), signifying that augmenting crop productivity substantially contributes to GHG emissions per unit of output. Under both investigated salinity regimes, the Global Warming Potential (GWP) displayed significant positive correlations with CO_2_, N_2_O, and CH_4_ emissions (P< 0.01). The correlation coefficient was the highest for CO_2_ (S1: r = 0.90; S2: r = 0.98, P< 0.001), which confirmed that CO_2_ served as the principal determinant of GWP variability within the cropping system. Furthermore, GHGI was significantly correlated with both GWP and CO_2_ (all r > 0.85, P< 0.001), substantiating the close linkage between GHGI and the cumulative carbon emission load during agricultural production.

Additionally, Soil Organic Carbon (SOC) and Soil Organic Carbon Storage (SOCS) exhibited significant negative correlations with all measured greenhouse gas indicators (P<0.05). The most substantial inverse correlation was detected between SOC and GHGI (S1: r = -0.75; S2: r = -0.89, P< 0.001), suggesting that elevated soil organic carbon content resulted in lower greenhouse gas emission intensity relative to crop yield. Concurrently, SOCS displayed a highly significant positive correlation with SOC (S1: r = 0.90; S2: r = 0.94, P< 0.001), reinforcing the conclusion that biochar application effectively enhanced soil organic carbon levels by augmenting soil carbon sequestration. Consequently, the judicious application of biochar to optimize crop yield and soil carbon pool status has a pivotal ecological function in curbing agricultural GHG emissions in saline-alkali environments.

### 3.5 Response optimization analysis of biochar application on yield, GWP, and SOCS

**Figure 5:**
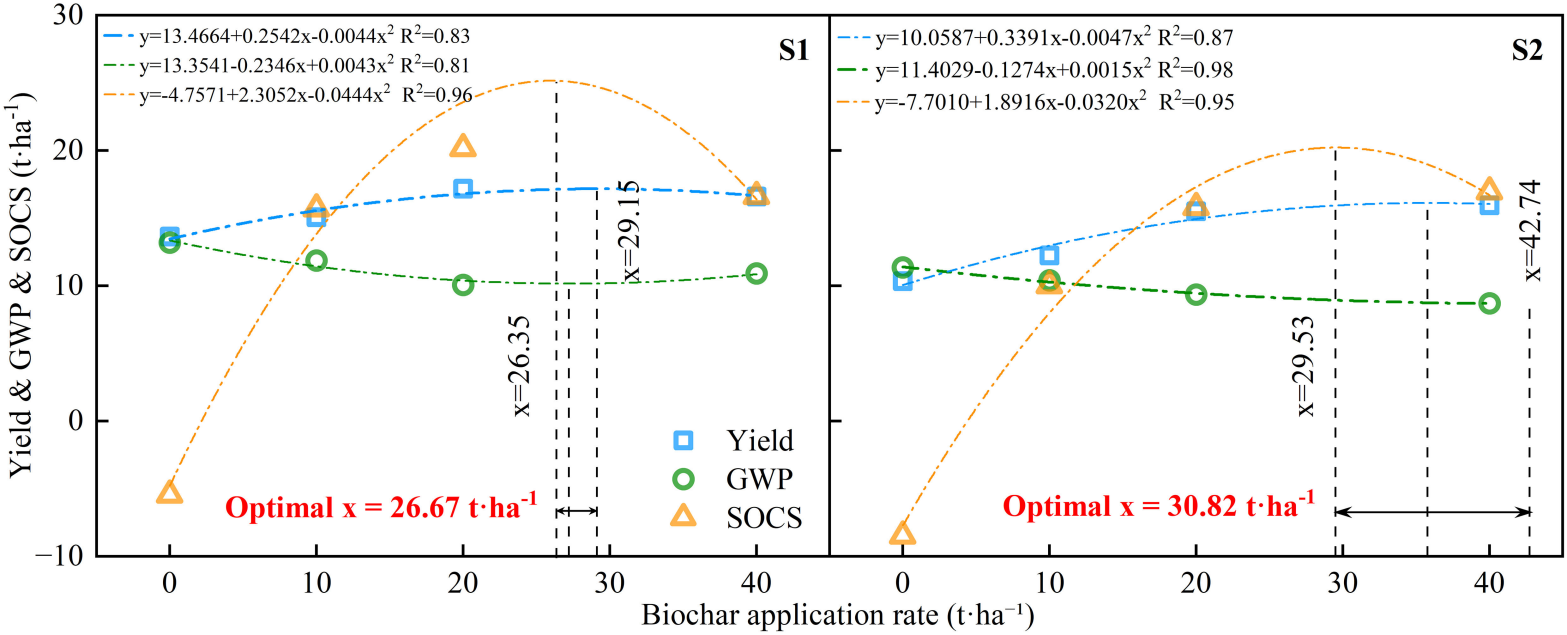
Response optimization of yield, global warming potential (GWP), and soil organic carbon sequestration (SOCS) to biochar application rates under different saline conditions (S1, slightly saline; S2, moderately saline) during 2022-2023.

An optimization analysis examining the responses of Yield, GWP, and SOCS to biochar application (**Figure 5**) revealed that the optimal application rates were dependent on soil salinity levels. Under mildly saline-alkali conditions (S1), an application rate of 26.67 t·ha^-1^ delivered the synergistic advantages of maximizing grain productivity, minimizing global warming potential (GWP), and maximizing soil organic carbon storage (SOCS). Conversely, under moderately saline-alkali conditions (S2), the corresponding optimal application rate was determined to be 30.82 t·ha^-1^.Furthermore, the derived fitted response curve equations demonstrated that under S1 conditions, SOCS gradually declined when the biochar application rate surpassed 29.15 t·ha^-1^, indicating that excessive biochar inputs were not conducive to further augmentation of soil carbon sequestration. Under S2 conditions, the rate required to maximize SOCS was 42.74 t·ha^-1^, which significantly exceeded the optimal rate established for balancing yield and GWP (30.82 t·ha^-1^). This outcome suggests that attaining superior soil carbon storage in moderately saline-alkaline environments necessitates a substantially greater input of biochar. Hence, to integrate yield, ecological, and environmental benefits, an application rate of approximately 26.67 t·ha^-1^ is recommended for mildly saline-alkali soils, whereas a rate of approximately 30.82 t·ha^-1^ is deemed more appropriate for moderately saline-alkali soils.

## 4 Discussion

### 4.1 Effects of biochar on greenhouse gas emissions, SOC, and crop yield

The findings of this study affirm that judicious biochar application substantially reduced CO_2_, N_2_O emissions while concurrently augmenting net CH_4_ uptake, demonstrating a pronounced synergistic mitigation effect under field conditions. The principal mechanisms are attributable to the inherent properties of biochar, including its porous structure and high specific surface area. These characteristics improve soil aeration and gas diffusivity, disrupting the contiguous anaerobic microsites required for methanogenesis (Nan et al., 2021). Simultaneously, these pores provide favorable habitats for methanotrophs, thereby suppressing CH_4_ production and promoting its oxidation (Shakoor et al., 2021). Furthermore, the physical adsorption capacity of biochar may increase the residence time of CH_4_ in these microenvironments, thereby enhancing its utilization efficiency (Ko et al., 2023; Kubaczyński et al., 2022). The consistently negative CH_4_ fluxes observed throughout the growing season indicate that maize fields under saline-alkali conditions function as weak carbon sinks, a capacity that is significantly strengthened by biochar application (Tao et al., 2023). Notably, CH_4_ absorption in 2023 was 1.6 times greater than that in 2022, a phenomenon likely attributable to shifts in the methanogen to methanotroph population balance as the biochar aged (Wang et al., 2018).

Regarding CO_2_ emissions, the recalcitrant aromatic structure of biochar confers high stability and resistance to microbial decomposition (Wakudkar and Jain, 2022). Biochar constitutes an exogenous carbon input and appears to slow the decomposition rate of native soil organic carbon (Wu et al., 2023a; Zewei et al., 2021), thus suppressing CO_2_ release from microbial respiration. Concurrently, biochar promotes the formation of organo-mineral complexes, which afford physical protection to organic matter and further curtail carbon mineralization losses (Yang et al., 2021). Although some studies have reported that biochar degrades more readily in acidic soils, potentially increasing CO_2_ emissions (Sheng and Zhu, 2018), the alkaline soil conditions in this study likely facilitated an abiotic sequestration pathway. A portion of mineralized CO_2_ may have precipitated and fixed as carbonates, resulting in lower net emissions (Linjie et al., 2021; Luo et al., 2016). Notably, under S1 conditions, the B40 treatment accelerated native soil organic carbon mineralization, suggesting that excessive biochar application can stimulate microbial activity by increasing carbon substrate availability and soil aeration, leading to diminishing marginal returns (Ren et al., 2025). Moreover, the significantly lower overall CO_2_ emissions in 2023 compared to 2022 suggest that the aging process depleted the labile carbon fraction in the biochar, reducing the release of volatile components and subsequent microbial respiration (Rahman et al., 2021).

N_2_O emissions were principally driven by nitrogen fertilization and irrigation events (Liu et al., 2011; Ma et al., 2025), with flux peaks occurring 4–5 days post-application. This study found that N_2_O emissions decreased significantly with increasing biochar application rate (Shi et al., 2021). This effect is likely due to the physicochemical adsorption of mineral nitrogen precursors such as NH_4_^+^-N and NO_3_^-^-N by biochar (Abbruzzini et al., 2019). In parallel, biochar improves soil aeration (Hu et al., 2024), and the resultant higher oxygen concentrations limit the incomplete reduction of nitrate during denitrification, thereby reducing N_2_O production in favor of complete reduction to dinitrogen gas (Song et al., 2019). Biochar also helps reshape microbial niches by enhancing soil pH buffering capacity and providing stable carbon sources and habitable micropores, which further suppresses N_2_O emissions (Bolan et al., 2022). The most stable mitigation effects were observed in the B20 treatment under S1 and B40 treatment under S2. The notable decrease in cumulative N_2_O emissions in the second year aligns with reports of enhanced inhibition of nitrogen transformation by aged biochar (Wang et al., 2024a).

As an organic amendment, biochar significantly increased soil organic carbon (SOC) content, with the most pronounced effects observed in the 0–20 cm soil layer. This increase reflects both the direct contribution of biochar’s substantial carbon input (Chagas et al., 2022) and its indirect role in inhibiting native organic matter mineralization and enhancing the physical protection of organic carbon within soil aggregates (Zhang et al., 2023a; Zhou et al., 2023). Under certain conditions, biochar can induce a negative priming effect on the SOCS through competitive microbial interactions (Chen et al., 2019; Yang et al., 2022). Concurrently, biochar application increases the adsorption of nutrient ions, which improves nitrogen use efficiency (Xia et al., 2023), thereby significantly enhancing maize yields. This study demonstrated that biochar application increased yield by 10.47%–54.21% and reduced GWP by 8.62%–24.12%, culminating in effective reductions in both GHGI and NGHGE (**Table 1**). In summary, the appropriate biochar application achieved a nexus of concurrent benefits, including emission reduction, carbon sequestration, and yield enhancement.

### 4.2 Moderating effect of salinity on biochar response

Pronounced divergences in the effects of biochar were observed across soils with varying salinity levels. Considering the differential impacts of salinity on CO_2_, CH_4_, N_2_O production, the resultant Global Warming Potential (GWP) was 7.41%–20.98% lower under S2 conditions than under S1 (**Table 1**). Concurrently, the Soil Organic Carbon Storage (SOCS) increased (**Figure 3**). These disparities were primarily attributable to intrinsic differences in physicochemical properties, water-salt distribution, and microbial activity between the two soil types. Soil S2 was characterized by lower organic matter content and elevated salt stress when compared with S1. This characteristic engendered a greater reliance on exogenous carbon sources and soil amendments, a condition that enabled biochar to manifest a more pronounced ecological restoration potential within S2 environments.

With respect to greenhouse gases, elevated salinity typically diminishes CH_4_ emissions across diverse ecosystems (Hu et al., 2017). This suppression mechanism is predominantly ascribed to high salinity enhancing sulfate reducibility by increasing SO_4_^2-^ availability, which significantly impedes methanogen activity (Haj-Amor et al., 2022) and consequently reduces soil CH_4_ fluxes. Conversely, soil salinity constrains CO_2_ production by suppressing microbial activity through reduced osmotic potential and increased ion toxicity within the soil solution (Yan et al., 2015). Consequently, consistently higher CO_2_ emissions were observed in S1 than those in S2 in the present investigation. In contrast, the elevated salt ion concentration inherent to S2 conditions induced osmotic stress in the microbial community (Polonenko et al., 1981). This stress inhibits the activity of specific nitrifying bacteria while concurrently promoting denitrification processes (Chen et al., 2022), which resulted in augmentedN_2_O emissions. Biochar application mitigated these adverse effects by enhancing pH buffering capacity (Liu et al., 2025), providing stable carbon substrates within habitable microbes and facilitating the reshaping of microbial niches (Bolan et al., 2022). This ameliorative effect is particularly important in salt-stressed conditions.

S2 exhibited a superior capacity for organic carbon sequestration. This was principally attributed to the intrinsic inhibitory influence of saline-alkaline environments on microbial decomposition, which decelerates the mineralization rate of organic matter and extends the carbon residence time (De Souza Silva and Fay, 2012; Rath and Rousk, 2015). Concomitantly, high-salinity conditions are known to attenuate the loss of dissolved organic carbon and augment the efficiency of physical carbon protection, thereby synergistically increasing SOCS levels (Mavi et al., 2012). Furthermore, saline-alkali soils frequently suffer from structural instability caused by colloid dispersion and rapid water-salt flux coupled with inadequate water-air regulation capacity (Xu et al., 2023). This instability amplifies the marginal benefits of biochar applications. The inherent porous structure and adsorptive characteristics of biochar offer great potential for improving the aggregate structure (Islam et al., 2021) and fostering micro-environmental stability (Duan et al., 2021; Huang et al., 2024). Consequently, its beneficial effects on soil physicochemical properties and carbon cycling processes were more pronounced in S2 (Wu et al., 2024b). Given the differential responses observed under varying salinity levels, the application rate of B20 was determined to be optimal for mildly saline-alkali soils, whereas a higher application rate of B40 was necessary for moderately saline- alkaline soils to achieve maximum ecological and productivity outcomes. These results provide a robust theoretical foundation for precise nutrient management and soil amelioration in saline-alkali lands. They also accentuate the extensive application prospects of biochar in promoting sustainable agricultural transformation within arid saline-alkali regions.

### 4.3 Application strategy and practical implications under multi-objective optimization

The relationships between Yield, GWP, and SOCS were nonlinear (**Figure 4**) and encompassed intricate synergies and trade-offs. As an exogenous carbon amendment, biochar augments soil carbon storage while concurrently modifying soil physicochemical properties and the composition of microbial functional groups (Rechberger et al., 2017; Singh et al., 2022). These modifications further influence nitrogen transformation processes and crop rhizosphere dynamics (Xia et al., 2025), resulting in non-uniform responses across various target variables (Khan et al., 2023; Pang et al., 2022). Consequently, the integration of multi-objective optimization methodologies into the design of biochar application strategies facilitates the identification of an optimal equilibrium among multidimensional ecological benefits. .

The findings indicated that an application rate of 26.67 t·ha^-1^ achieved a simultaneous maximization of yield and control of greenhouse gas emissions, in mildly saline-alkali soil (S1). In moderately saline-alkali soil (S2), however, the required rate was elevated to 30.82 t·ha^-1^ to realize superior comprehensive benefits. This pattern reflects the differential regulatory capacity exerted by the saline-alkali soil environment on the mechanisms of biochar action. Under conditions of lower salinity, biochar predominantly enhanced crop yield indirectly by promoting root growth and improving nutrient availability, although with comparatively moderate marginal gains observed in greenhouse gas mitigation and carbon sequestration (Wan et al., 2023). Conversely, under elevated salinity conditions, biochar showed more pronounced potential for carbon sequestration enhancement and emission reduction. This outcome was attributed to its robust effects on pH buffering, effective water-salt regulation, and restoration of microbial diversity (Wu et al., 2024b; Yang et al., 2025). Although biochar generally benefits saline-alkali soils, excessively high application rates may cause adverse effects (Lin et al., 2024; Wu et al., 2024a). Over-application can increase soil pH and electrical conductivity, and create nutrient immobilization or imbalance, thereby constraining root uptake and suppressing gas-mitigation or yield benefits (Li et al., 2018; Wei et al., 2020). These potential trade-offs help explain why the response optimization identified intermediate optima (26.67 t·ha^-1^ for S1 and 30.82 t·ha^-1^ for S2), rather than a monotonic improvement with increasing biochar rate.

Furthermore, the optimization analysis revealed that the application rates required to maximize yield, minimize GWP, and maximize SOCS did not fully coincide. This observation indicates the inherent tensions among objective functions within agricultural ecosystems. Such tension typically arises because the excessive application of biochar, while capable of augmenting SOCS, may concurrently stimulate N_2_O emissions (Liu et al., 2019). Conversely, insufficient application, while potentially favorable for emission reduction, constrains the attainment of optimal crop productivity (Prapagdee and Tawinteung, 2017). On a practical level, the differentiated application rates proposed herein facilitate zonal application and precision management within saline-alkali lands (He et al., 2020; Qiu et al., 2025). This approach offers a scientifically grounded pathway toward attaining carbon neutrality objectives in regional agricultural systems (Zhang et al., 2020; Zhu et al., 2020).

## 5 Conclusions

This study demonstrated that biochar application significantly augmented crop yield, reduced greenhouse gas emissions, and increased organic carbon storage in saline-alkali soils. Under both mild (S1) and moderate (S2) saline-alkali conditions, a biochar application rate of 20 t·ha^-1^ (B20) achieved an optimal equilibrium between yield enhancement and environmental benefits. This rate effectively mitigated GWP, GHGI, and NGHGE, while substantially increasing SOC and SOCS. Correlation analysis confirmed significant negative correlations between soil carbon indicators and greenhouse gas emissions. Furthermore, response optimization analysis identified the ideal biochar application rates as 26.67 t·ha^-1^ for S1 and 30.82 t·ha^-1^ for S2. These findings indicate that judicious biochar application is an effective strategy for enhancing the carbon sink potential of saline-alkali farmlands, mitigating climate-related risks, and promoting sustainable agricultural development. Future research should prioritize the assessment of long-term effects and economic feasibility to establish a comprehensive scientific basis for large-scale implementation of biochar on marginal land. 

## Data Availability

The original contributions presented in the study are included in the article/supplementary material. Further inquiries can be directed to the corresponding authors.
